# Evaluation of Apically Extruded Debris and Cleaning Efficiency of the Pediatric Rotary File System and the Manual Nickel-Titanium File System

**DOI:** 10.7759/cureus.36603

**Published:** 2023-03-23

**Authors:** Abrar Habib, Enas Hegazi, Shaimaa Mahfouz

**Affiliations:** 1 Faculty of Dentistry, Suez Canal University, Ismailia, EGY; 2 Pediatric Oral Biology Department, Faculty of Dentistry, Suez Canal University, Ismailia, EGY; 3 Pediatric Dentistry, Preventive Dentistry and Dental Public Health Department, Faculty of Dentistry, Suez Canal University, Ismailia, EGY

**Keywords:** pediatric rotary file system, manual file system, pediatric, ni-ti rotary files, rotary file system

## Abstract

Background

This study aimed to assess and compare the Kidzo pediatric rotary file system with the manual nickel-titanium (NiTi) K-file system used for preparing the root canal of primary mandibular second molars by measuring the total apically extruded debris utilizing a sensitive microbalance and assessing cleaning efficiency using a scanning electron microscope.

Methodology

A total of 46 mandibular second primary molars were instrumented using a pediatric rotary system (Elephant Kidzo, India) and a manual NiTi K-file system (Endostar, Poldent, Poland). The debris released from the apex was retrieved and dried in Eppendorf tubes that were weighed beforehand. The total extruded debris was determined using a digital electronic scale, following which the molar roots were vertically sectioned, and the canal walls were investigated at the apical, middle, and coronal levels by a scanning electron microscope for debris and smear layer.

Results

The Kidzo pediatric rotary file system extruded a lesser amount of debris than the manual Endostar file system, but the difference was statistically not significant. Regarding cleaning efficiency, the debris originating in the apical and middle regions by employing the rotary filing technique was significantly less (p < 0.05); however, at the coronal level, there were no notable differences.

Conclusions

The Kidzo pediatric rotary system produced less apically extruded debris than the manual system and demonstrated better cleaning efficiency.

## Introduction

A pulpectomy is a root canal technique designed for severely damaged or necrotic primary teeth because of caries or trauma [1]. It involves the mechanical or chemical excision of the severely infected or necrotic radicular pulp tissue, and then filling the root canal with a resorbable substance [2]. For primary teeth, the purpose of pulpectomy techniques is mainly to remove organic debris. This can be accomplished with manual or rotary nickel-titanium (NiTi) tools. However, the traditional manual approach is time-consuming and frequently makes the operator and the child exhausted [3].

The development of the NiTi rotary instrument improved primary tooth endodontics to become more efficient, easier, and quicker than manual tools. Different pediatric rotary systems have been developed and are widely used in preparing deciduous teeth root canals. NiTi file designs and great flexibility enable them to precisely follow the original root canal route, particularly in curved canals, and significantly decrease technical errors such as ledges, apical transport, and over-instrumentation [4]. Hence, the goal of our research is to assess and compare the Kidzo pediatric rotary system and the manual NiTi system by measuring apically extruded debris and cleaning efficiency using scanning electron microscopy (SEM).

## Materials and methods

This in vitro study was performed in partnership between the Department of Pediatric and Preventive Dentistry and the Department of Oral Biology, Faculty of Dentistry, Suez Canal University after obtaining approval from the Research Ethical Committee (REC), Faculty of Dentistry, Suez Canal University (approval number 334/2021 dated 11/04/2021). A total of 46 unidentified extracted human primary mandibular second molars were collected for evaluating apically extruded debris and cleaning efficiency according to the following inclusion criteria: molars having a minimum of two-thirds of the distal roots, no internal and/or external pathologic root resorption (RR), and no noticeable root caries, fractures, or cracks. Periapical radiographs were obtained to exclude molars with internal resorption or intracanal calcification [5]. Only molars with one apical foramen and one canal distal root were involved in the study. Root morphology was investigated using buccal and proximal radiographs, as well as by examining the root apical area using a stereomicroscope (Expert DN; Muller Optronic, Erfurt, Germany).

Sample preparation

All selected molars were sterilized by soaking in 0.5% chloramine solution at 4°C for 48 hours, followed by a 24-hour rinse under running water. Caries or old restorations were removed from all molars. The roof of the pulp chamber was removed and access to the pulp was gained using carbide burs (Kerr, USA) in a high-speed handpiece (COXO, China) using a water cooling system. To maintain a standard mean length of 10 mm for each root, the teeth crowns were partly cut using a diamond disc (KG Sorensen, Sao Paulo, Brazil) near the cementoenamel junction.

Canal orifices were located with an explorer, following which each molar was sectioned at the furcation area into two separate mesial and distal roots using a dental surgical bur with a water coolant. Only distal roots were included in this study and the mesial roots were discarded. A glide path using a #20 K-file (Dentsply/Maillefer, Switzerland) was established, and the apical patency was verified. The working length, which was 1 mm less than the actual one, was measured by slipping a #10 K-file (Dentsply/Maillefer, Switzerland) into the root canal until the appearance of its head at the apical foramen under a magnifying loupe [6].

Sample grouping

The selected 46 distal roots of lower primary second molars were numbered from 1 to 46 and then randomly divided (using the website https://www.randomizer.org) into three groups in accordance with canal preparation techniques as follows: group I: six without any canal preparation (the control group); group II: 20 samples prepared using the Elephant Kidzo rotary system; and group III: 20 samples prepared using the manual Endostar NiTi K-file system.

Elephant Kidzo Rotary Instrumentation (Group II)

In group II, distal roots were prepared using the Kidzo pediatric rotary file system (Elephant, India). First, K-files #10 and #15 (Dentsply/Maillefer, Switzerland) were employed to negotiate the canal, and then the rotary files were utilized following the manufacturer’s guidelines in a torque-controlled endodontic handpiece (SNAIL, China) at a speed of 350 rpm in the following order: size 25/.04, size 30/.04, size 30/.06. Each file was used with minimum apical pressure in a light brushing motion [7].

Endostar Manual NiTi Instrumentation (Group III)

In group III, distal roots were prepared using the manual NiTi K-file system (Endostar, Poldent, Poland) at the working length with sizes 20, 25, 30, and 35 sequentially. Each file was moved in the canal for about 10 seconds, removed, cleaned with sterile gauze, and reinserted if needed to achieve the working length, following which the next size was used [8].

During instrumentation in both groups, all distal root canals in groups II and III were rinsed with 2.5% NaOCl (3 mL) (Dharma, Calix, USA) in between each file size and 29‑gauge double side‑port needles (NaviTip Sideport; Ultradent, USA) were used for the final flushing with 17% ethylenediaminetetraacetic acid (1 mL) (PREVEST DenPro, India), 2.5% NaOCl (3 mL), and saline (3 mL) (0.9% sodium chloride) (Otsuko, Egypt) [9].

After each retrieval, the files were examined for deformation using a portable magnifying lens under illuminated light. The deformed files were discarded, whereas the non-deformed files were thrown after their second use.

The main operator prepared no more than five roots at a time to prevent operator fatigue-related errors. All root canal preparations were performed by one practitioner complying with the manufacturer’s recommendations, while the evaluation of debris was done by another practitioner blinded to all treatment groups.

Debris collection

To measure the extruded debris in this study, the Myers and Montgomery [10] method was applied. The weight of 40 Eppendorf tubes was measured using a digital microbalance (Sartorius, Germany). Each tube weight was taken three times and the mean was calculated for each tube. After removing the stoppers of Eppendorf tubes, holes were drilled into them. The teeth were put through the caps up to the cementoenamel junction and glued with cyanoacrylate (Pattex Super Glue; Türk Henkel, Inc., Istanbul, Turkey) to block the irrigation solution from leaking out. To regulate the air pressure in and out of the tube, a needle was put through its cap. To prevent examiner bias, all tubes were wrapped with aluminum foil. They were decoded with nail polish according to the type of preparation. Group II (rotary group) was coded with blue color, and group III (manual group) was coded with pink color.

Debris assessment

After the canal preparation for groups II and III, the debris stuck to the exterior surface of the root apex was retrieved by rinsing it with 1 mL sterile saline, following which the roots were removed from the tubes. The tubes were kept for five days at 70°C in an incubator to remove any moisture [11]. After complete evaporation, each vial with salt residues and dry debris was weighed with 10-4 precision thrice (w2). The dry debris mean weight (w3) was evaluated by deducting the initial weight of the empty Eppendorf tube (w1) from the total weight (w2) as follows: w3 = (w2 - w1).

Measuring cleaning efficacy using scanning electron microscopy

A groove was cut longitudinally using a taper fissure diamond bur (#846) for all distal roots in the three groups. The last part of the root was separated with a chisel. The root halves were cleansed of scraping debris with water and dried by blowing air for three seconds. One half from each separated root was randomly selected using a coin flip for further SEM analysis [12].

Each specimen was coated with a thin layer of gold (10-30 mm) and mounted on aluminum stubs using a conductive paste (carbon paste) and placed in the JFC-1100E ion sputtering device. For each canal, the entire surface and each section (apical, middle, and coronal) were inspected at a magnification of ×500 for analysis. Blinded coated root halves were evaluated by an experienced and calibrated examiner (EH).

Quantitative measurements were made for the debris and smear layer on the canal walls and images were analyzed independently by three investigators. The group that each sample belonged to as well as the absence, partial, or total presence of debris and/or smear layer were hidden from the investigators. Based on the Hulsmann scoring system for smear layer and debris, the evaluators gave each image a score between 1 and 5 with respect to smear layer and debris, as described below. The debris scale is presented in Table 1, and the smear layer scale is presented in Table 2.

**Table 1:** The debris scale.

Score	Description
Score 1	Clean wall and contains minor debris only
Score 2	Few masses of debris
Score 3	Lesser than 50% of the wall is covered by several debris clusters
Score 4	Above 50% of the wall is covered by debris
Score 5	Whole or almost whole layer of debris covering the wall

**Table 2:** The smear layer scale.

Score	Description
Score 1	Absence of the smear layer and all dentinal tubules are opened
Score 2	A small quantity of the smear layer and some dentinal tubules are opened
Score 3	The wall of the root canal is coated with a homogenous smear layer and only a few dentinal tubules are opened
Score 4	The wall of the root canal is completely wrapped by a homogenous smear layer and the absence of opened dentinal tubules
Score 5	The wall of the root canal is completely coated with a heavy, homogenous smear layer

Statistical analysis

Independent-sample t-test was employed to statistically analyze the data at a significance level of p >0.05. Data were analyzed using SPSS version 24.0 software (IBM Corp., Armonk, NY, USA).

## Results

Evaluation of extruded debris

**Figure 1:**
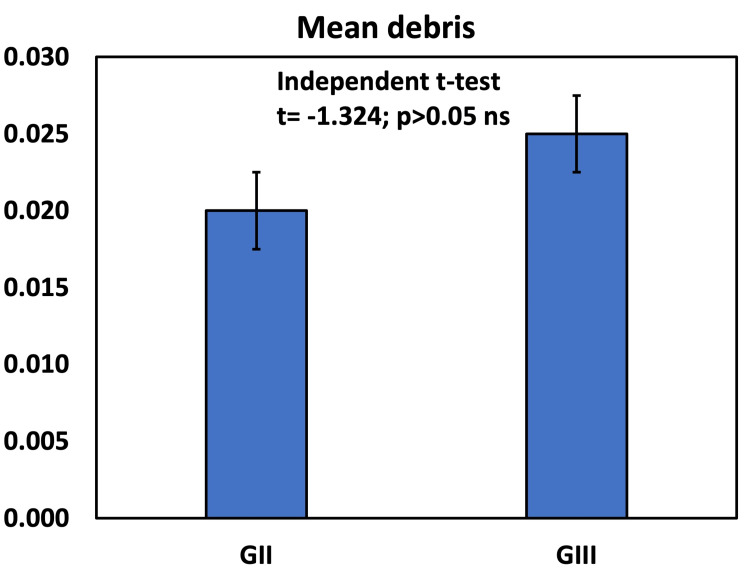
Bar chart representing the mean (±standard deviation) debris from groups II and III.

All study instruments produced apically extruded debris. According to the statistical findings, the rotary group (II) extruded a slightly smaller amount of debris than the manual group (III). However, there was no statistically significant difference between the groups (t = -1.324; p > 0.05) (Figure 1).

Measurement of cleaning efficiency

Debris Scale

**Figure 2:**
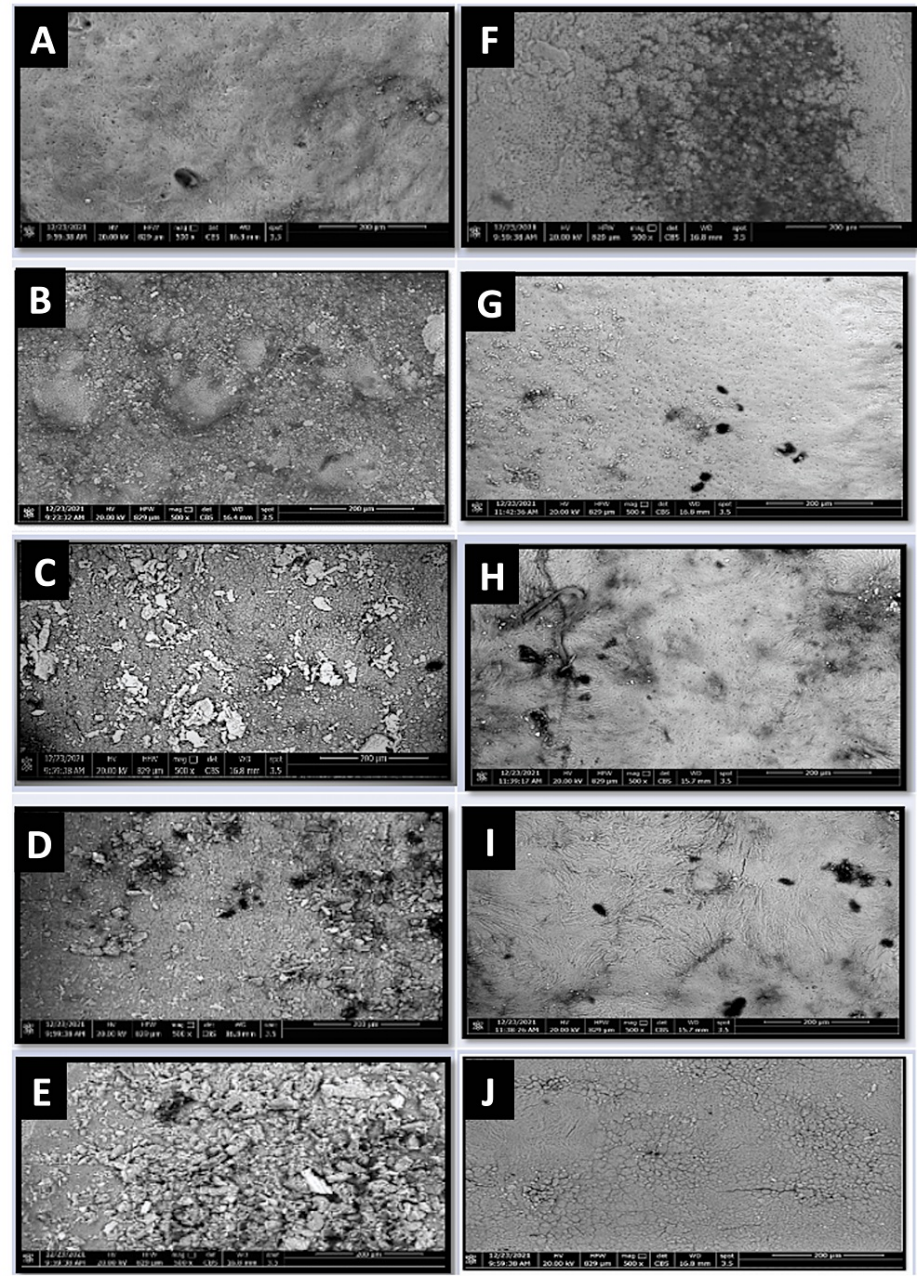
Scanning electron micrographs of the root canal dentin surface instrumented with rotary system (group II) showing different scores of debris (A-E) and smear layer (F-J). Scores of debris from A to E represent scores from 1 to 5, and from F to J represent smear layer scores from 1 to 5.

**Figure 3:**
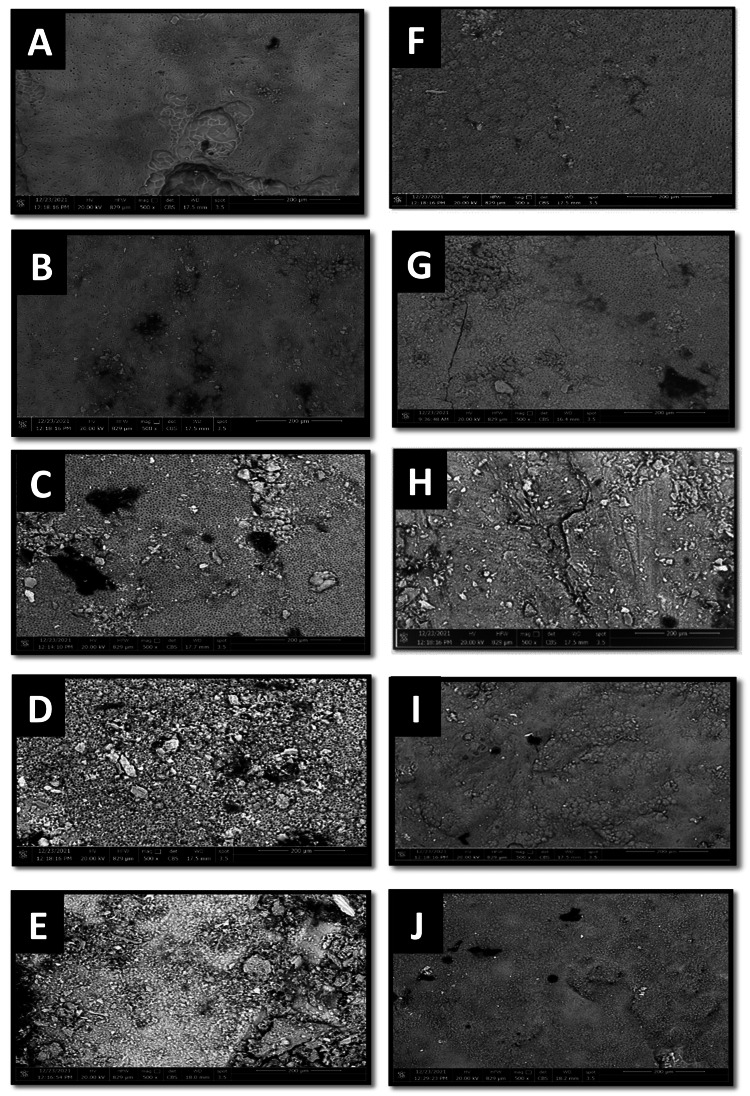
Scanning electron micrographs of the root canal dentin surface instrumented with rotary system (group III) showing different scores of debris (A-E) and smear layer (F-J). Scores of debris from A to E represent scores from 1 to 5, and F to J represent smear layer scores from 1 to 5.

In group I (control group), there was no significant difference (p > 0.05) among scores at the apical (p = 0.607), middle (p = 0.607), and coronal (p = 1.00) levels. In group II (rotary group), there was a significant difference among scores in the apical (p = 0.022) and middle (p = 0.019) levels; however, scores at the coronal level were non-significant (p = 0.199). In group III (manual group), the difference was non-significant among scores at the apical (p = 0.478), middle (p = 0.079), and coronal (p = 0.308) levels (Table 3). Table 3 presents a comparison between the apical, middle, and coronal thirds for all groups regarding the debris score. The debris scores of group II are presented in Figure 2 (Panels A-E), and the debris scores for group III are presented in Figure 3 (Panels A-E).

**Table 3:** Comparison between apical, middle, and coronal thirds for all groups regarding debris score. Data are presented as frequency n, %. *, **, ***: significant at p < 0.05, <0.01, <0.001, respectively; ns: non-significant at p > 0.05.

Level	Group	Debris score frequency, n (%)											Chi-square
		Score 1		Score 2		Score 3		Score 4		Score 5		Total	
		n	%	n	%	n	%	n	%	n	%		P-value
Apical	Group I	0	0.0	1	16.7	3	50.0	2	33.3	0	0.0	6	0.607 ns
	Group II	6	30.0	12	60.0	2	10.0	0	0.0	0	0.0	20	0.022 *
	Group III	6	30.0	5	25.0	4	20.0	4	20.0	1	5.0	20	0.478 ns
	Total	12	26.1	18	39.1	9	19.6	6	13.0	1	2.2	46	0.001***
Middle	Group I	0	0.0	1	16.7	3	0.0	2	0.0	0	0.0	6	0.607 ns
	Group II	2	10.0	9	45.0	8	40.0	1	5.0	0	0.0	20	0.019 *
	Group III	4	20.0	6	30.0	9	45.0	1	5.0	0	0.0	20	0.079 ns
	Total	6	13.0	16	34.8	20	43.5	4	8.7	0	0.0	46	0.001***
Coronal	Group I	0	0.0	0	0.0	3	0.0	3	0.0	0	0.0	6	1.00 ns
	Group II	3	15.0	7	35.0	3	15.0	6	30.0	1	5.0	20	0.199 ns
	Group III	0	0.0	7	35.0	7	35.0	4	20.0	2	10.0	20	0.308 ns
	Total	3	6.5	14	30.4	13	28.3	13	28.3	3	6.5	46	0.007**
Friedman’s test		<0.001***											

Smear Layer Scale

In group II (rotary group), the difference among scores was non-significant at the apical (p = 0.423), middle (p = 0.406), and coronal (p = 0.736) levels. In group III (manual group), the difference among scores was non-significant at the apical (p = 0.736), middle (p = 0.343), and coronal (p = 0.974) levels (Table 4). The smear layer scores of group II are presented in Figure 2 (Panels F-J), and the smear layer scores for group III are presented in Figure 3 (Panels F-J).

**Table 4:** Comparison between smear layer scores in the apical, middle, and coronal thirds in groups II and III. Data presented are presented as frequency n, %. *, **, ***: significant at p < 0.05, <0.01, <0.001, respectively; ns: non-significant at p > 0.05.

Level	Group	Smear layer scale frequency n (%)										Chi-square
		Score 1		Score 2		Score 3		Score 4		Score 5		
		n	%	n	%	N	%	n	%	n	%	P-value
Apical	Group II	7	35.0	5	25.0	6	30.0	2	10.0	0	0.0	0.423 ns
	Group III	4	20.0	6	30.0	4	20.0	4	20.0	2	10.0	0.736 ns
	Total	11	27.5	11	27.5	10	25.0	6	15.0	2	5.0	0.123 ns
Coronal	Group II	5	25.0	5	25.0	3	15.0	5	25.0	2	10.0	0.736 ns
	Group III	4	20.0	3	15.0	5	25.0	4	20.0	4	20.0	0.974 ns
	Total	9	22.5	8	20.0	8	20.0	9	22.5	6	15.0	0.971 ns
Middle	Group II	6	30.0	4	20.0	6	30.0	2	10.0	2	10.0	0.406 ns
	Group III	5	25.0	7	35.0	4	20.0	2	10.0	2	10.0	0.343 ns
	Total	11	27.5	11	27.5	10	25.0	4	10.0	4	10.0	0.406 ns
Friedman’s test		P = 0.001***										
Source		F			P-value							
Corrected model		38.56			0.004**							
Intercept		937.31			<0.001***							
Groups		31.05			<0.001***							
Level		2.85			0.415 ns							
Group* level		2.8			0.784 ns							

## Discussion

In this study, the apically extruded debris was collected using the Myers and Montgomery [9] method. In contrast, some studies have suggested slight modifications in the collection method. According to these studies, the extruded debris weight may be impacted by contact with moist or greasy fingertips. Therefore, these studies avoided the chance of contamination during the entire experiment by preventing the operator’s fingertips from making direct contact with the equipment [13,14].

To our knowledge, to date, no study has investigated apical extrusion during instrumentation with the Kidzo pediatric rotary device. The results of this study show that the pediatric rotary system extrudes a lesser quantity of debris than the manual method but without any notable significant difference.

Our finding is in the line with that of Nanavati et al. [15] who found that throughout pulpectomy, pediatric rotary files extruded considerably less quantity of apical debris than manual files when measuring apically extruded debris of distal roots of 60 primary lower molar teeth by utilizing two types of rotary files and hand files, namely, Prime Pedo™ pediatric rotary files, DXL-Pro Pedo™ pediatric rotary files, and hand K-files.

On the other hand, rotary files extruded significantly less debris compared with manual files when assessing the apically extruded debris through instrumentation of 45 primary canines using three different types of endodontic files, namely, manual K-files, rotary Kedo-S files, and XP-endo shaper files [16].

The study findings showed that all equipment and tools cause debris to be extruded at the apex. According to previous research [17,18], no approach can entirely prevent debris extrusion, and our study findings are consistent with these previous findings.

In this study, SEM was used to evaluate the cleaning efficiency as it is more accurate in the evaluation of cleaning efficiency by two separate scoring systems; one for debris and the other for the smear layer [19]. This is in contrast with some previous studies [20,21] that evaluated the cleaning efficiency of endodontic files by inexpensive methods. They injected India ink into the root canals of selected specimens before preparation and then observed the canals under a stereomicroscope after complete instrumentation, and the scoring system depended on the amount of India ink remaining.

In this study, there were no detectable and statistically significant differences between any of the groups regarding debris removal in the coronal thirds of all instrumented root canals. However, in the middle and the apical thirds, a considerable difference was noted in the Kidzo group. This revealed that the rotary pediatric file produces cleaner root canal walls in the apical and middle thirds than manual instrumentation. On the other hand, this finding disagreed with that of Reddy et al. [22] who found that debris aggregation was more in the apical part than in the coronal and middle parts of the root canals prepared with all rotary files types using SEM examination when comparing cleaning efficacy of pro taper rotary files, K3 rotary files, and st-st manual files on 60 single-rooted human maxillary anterior teeth. They explained this by the anatomy of the root canal as the apical area is the narrowest part. Therefore, in this location, greater instrument contact generates more debris, and flushing cannot be performed effectively in this narrowest portion.

According to our findings, there was an increase in the mean smear layer in the manual group with no considerable differences between the two groups regarding the smear layer removal in all thirds (apical, middle, and coronal). This result implies that both rotary and manual instrumentation can produce clean root canal walls from the smear layer. This agrees with the findings of Reddy et al. [22] who found no notable difference in smear layer at any level among all groups when comparing the cleaning efficacy of pro taper rotary files, K3 rotary files, and st-st manual files on 60 single-rooted human maxillary anterior teeth.

However, our result disagreed with those of Manjunatha et al. [12] who found no considerable differences in the amount of smear layer for manual K-file and rotary ProFile at the cervical and middle thirds of the canals, but in the apical third, there was a highly significant difference, when they evaluated the cleaning efficacy of manual K flex files and rotary ProFile systems utilizing 30 single-rooted mandibular first premolars. Moreover, Elnagar et al. [23] using the Revo-S rotary system showed less smear layer than manual instrumentation at all root canal levels when comparing the cleaning efficiency of Revo-S rotary files and NiTi Flex K-file for root canal preparation of 30 human primary teeth with single root using SEM. In this study, the Kidzo pediatric file system was used in the instrumentation of primary molars and compared with the NiTi manual system regarding extruded debris and cleaning efficiency.

There are some limitations of this study. In the primary teeth that are characterized by fine and resorbing root canals, the pediatric file system should be used cautiously and at a controlled speed. Moreover, due to oddities caused by SEM specimen preparation, it is impossible to accurately measure the debris and smear layer depth. Finally, the time of instrumentation was not measured, which is important clinically to evaluate time consumption.

## Conclusions

According to the findings of this study, it can be concluded that the Kidzo pediatric rotary system produces less apically extruded debris than the manual NiTi system. It also displayed better cleaning efficiency than the manual NiTi system. Moreover, the Kidzo rotary system can be an alternative method for preparing root canals in primary teeth with less apically extruded debris and better cleaning efficiency. Based on the obtained results, the Kidzo rotary system is recommended as an alternate technique for preparing primary teeth root canals with less apically extruded debris and better cleaning efficiency.
